# Comparison of the APAS Independence Automated Plate Reader System with the Manual Standard of Care for Processing Urine Culture Specimens

**DOI:** 10.1128/spectrum.01442-22

**Published:** 2022-08-16

**Authors:** Megan Chiu, Peiting Kuo, Khrissa Lecrone, Andrew Garcia, Ruohui Chen, Natalie E. Quach, Xin M. Tu, David T. Pride

**Affiliations:** a Department of Pathology, UC San Diego Health, San Diego, California, USA; b Division of Biostatistics and Bioinformatics, Herbert Wertheim School of Public Health and Human Longevity Science, UC San Diego, San Diego, California, USA; c Department of Medicine, UC San Diego Health, San Diego, California, USA; Johns Hopkins Hospital

**Keywords:** urine cultures, automation, antimicrobial susceptibility testing, image processing, artificial intelligence, machine learning

## Abstract

Urine cultures are among the highest-volume tests in clinical microbiology laboratories and usually require considerable manual labor to perform. We evaluated the APAS Independence automated plate reader system and compared it to our manual standard of care (SOC) for processing urine cultures. The APAS device provides automated image interpretation of urine culture plate growth and sorts those images that require further evaluation. We examined 1,519 specimens over a 4-month period and compared the APAS growth interpretations to our SOC. We found that 72 of the 1,519 total specimens (4.74%) had growth discrepancies, where these specimens were interpreted differently by the APAS and the technologist, which required additional evaluation of plate images on the APAS system. Overall, there were 56 discrepancies in pathogen identification, which were present in 3.69% of the cultures. An additional pathogen was uncovered in a majority of these discrepancies; 12 (21.4%) identified an additional pathogen for the SOC, and 40 (71.4%) identified an additional pathogen for the APAS workflow. We found 214 (2.69%) antimicrobial susceptibility test (AST) discrepancies; 136 (1.71%) minor errors (mEs), 41 (0.52%) major errors (MEs), and 36 (0.45%) very major errors (VMEs). Many of the MEs and VMEs occurred in only a small subset of 13 organisms, suggesting that the specimen may have had different strains of the same pathogens with differing AST results. Given the significant labor required to perform urine cultures, the APAS Independence system has the potential to reduce manual labor while maintaining the identity and AST results of urinary pathogens.

**IMPORTANCE** Urine cultures are among the highest-volume tests performed in clinical microbiology facilities and require considerable manual labor to perform. We compared the results of our manual SOC workflow with that of the APAS Independence system, which provides automated image interpretation and sorting of urine culture plates based on growth. We examined 1,519 urine cultures processed using both workflows and found that only 4.74% had growth pattern discrepancies and 3.69% pathogen identification discrepancies. There was substantial agreement in AST results between workflows, with only 2.69% having discrepancies. Only 1.71% of the ASTs had mEs, 0.52% had MEs, and 0.45% had VMEs, with most of the MEs and VMEs belonging to a small subset of organisms. The APAS system significantly decreased manual urine culture processing, while providing similar results to the SOC. As such, incorporating such automation into laboratory workflows has the potential to significantly improve efficiency.

## INTRODUCTION

Urinary tract infections (UTIs) are the most commonly diagnosed bacterial infections we encounter (1). Consequently, urine cultures are among the highest-volume tests performed in most clinical microbiology laboratories (2). In the United States, these infections account for about 100,000 hospitalizations, 1 million emergency room visits, and 7 million office visits annually (3). UTIs can be caused by a myriad of bacterial pathogens, and there is some subjectivity in the interpretation of urine culture results. Consequently, diagnoses can be subject to variation among the technicians analyzing culture results (4). The gold standard for processing a urine test involves planting a urine specimen onto culture medium and enumerating the potential pathogens to determine whether there is sufficient growth to be reported. However, this process can be time-consuming and inefficient (5). Thus, automated systems can be effectively employed to reduce the manual labor involved in this process (6). The use of automated systems can free up valuable time for laboratory professionals to focus only on the clinically significant culture results that affect patient care, while reducing their efforts toward clinically insignificant growth. Reducing the time required to make a culture diagnosis is also important, as this can determine when the correct antibiotics may be used to treat the patient. Significantly expediting culture identifications could reduce the use of inappropriate antibiotics and a reliance on empirical antibiotics (7).

Automation has become more prevalent in clinical microbiology laboratories to help meet demands to reduce the hands-on labor necessary for processing specimens (8). To date, two major manufacturers, Copan Diagnostics and Becton, Dickinson and Company (BD), have provided laboratory automation systems—specifically, the Copan Walk-Away specimen processor (WASP) lab and the BD Kiestra system, respectively. The Copan WASP is designed to plate liquid specimens from a variety of transport devices, while the Copan WASPLab further integrates the WASP with plate incubation and high-resolution digital imaging/analysis driven by artificial intelligence (AI) algorithms (9). The BD Kiestra system inoculates specimens onto plates and provides incubation and AI-driven plate imaging/analysis (10). Although each of these systems decrease the workload on clinical staff and the time for processing most specimens, the resulting reliance on automation can reduce staff competency for performing manual workflows when necessary (11). The APAS Independence system, created by Clever Culture Systems, is an artificial intelligence-driven automated culture plate-reading system that can feed into existing laboratory infrastructure and provide a balance between manual and automated workflows. With the APAS system, laboratories can expedite the overall diagnostic process by reducing plate sorting times (processing up to 200 plates per hour) and allowing technicians to prioritize the analysis of positive cultures rather than evaluating the plates for all the negative cultures (4). The system functions by relying upon artificial intelligence to interpret growth patterns on urine plates and determine whether that growth may be clinically significant (12–14).

Conventional microbiology laboratories are highly reliant upon manual labor and the interpretative assessments of laboratory technicians (12). Unfortunately, many laboratories are experiencing labor shortages of trained and skilled staff, with an average vacancy rate of 7.2% among all laboratory departments and a vacancy rate of 7.26% across the western United States (15). The SARS-CoV-2 (severe acute respiratory syndrome coronavirus 2; COVID-19 [coronavirus disease 2019]) pandemic has also contributed to the labor shortage, since strict public health restrictions have affected many employees’ availability to work (16). With the high volume of specimens that need to be processed and diminished worker availability, automation systems have become more attractive options for clinical microbiology laboratories. Additionally, automation may provide financial relief for clinical microbiology labs because manual labor represents one of their greatest expenses (17).

The purpose of this study was to determine the impact of the APAS Independence system on urine cultures in our facility. We hypothesized that the inclusion of the APAS automation would result in substantial decreases in the hands-on time for processing cultures, while providing similar identification (ID) and antimicrobial susceptibility test (AST) results.

## RESULTS

### Study design.

We compared the results obtained using the APAS instrument to those from our standard of care (SOC) for processing urine cultures. The SOC involved plating 1 μL of urine specimens onto sheep’s blood agar and MacConkey agar plates using the Copan WASP automated plating instrument (18). The resulting streaked plates were incubated at 37 ± 2°C for approximately 18 to 24 h before being manually read for patterns of growth. Bruker MALDI-TOF technology was used to identify pathogens on plates with growth that indicated the potential presence of pathogens. Each microbe was then isolated so that antimicrobial susceptibility tests (ASTs) could be performed on the BD Phoenix M50 instrument, which uses different panels depending on the identity of the pathogen. The APAS workflow varied slightly from that of the SOC, since the plates were incubated for 18 h and immediately placed on the APAS instrument, which determined whether the patterns of growth indicated a need for further evaluation. The instrument sorted plates based on whether there was no growth, growth at an enumeration level of <10^4^ CFU/mL, or pure/predominant growth with an enumeration of ≥10^4^ CFU/mL. All other cultures not fulfilling these criteria were sorted for further review.

### Growth differences.

We examined 1,519 urine specimens over a period of 4 months. Each specimen was plated twice (separately for each workflow), and the resulting cultures were treated separately, with one set treated according to the SOC and the other directed toward the APAS workflow. We then compared the APAS results with those of the SOC to determine whether there were discrepancies in the patterns of growth between the two workflows. We found that of those 1,519 specimens, 1,461 (95.13%) had matching growth patterns between the SOC and the APAS instrument (Table 1). These results included many plates with either no growth, mixed urethral flora (MUF), or potential pathogens. We identified 74 total growth discrepancies of the 1,519 total specimens (4.87%), where differences in interpretations of growth patterns were observed among the workflows. While the observed growth discrepancies represent less than 5% of the cultures performed, they do represent a significant portion of the cultures (95% CI, 3.8% to 6.1%; Table 1). Of those 74 observed differences in growth interpretations, we found that 72 (4.74%; 95% CI, 3.7% to 5.9%) did not result in the identification of clinically significant pathogens. However, 2 (0.13%; 95% CI, 0% to 0.5%) did result in the identification of pathogens that could have affected clinical care. In 1 of these 2 samples, the SOC workflow identified at least three different colony morphologies from a typical site and determined the culture to be mixed urethral flora. The APAS workflow for this same specimen identified the pathogen Proteus mirabilis. In the other discrepant clinically significant specimen, the SOC identified Escherichia coli, whereas fewer than 10 colonies were seen on the study plate. Consequently, the study designation for this plate was “no significant growth” (NSG). The >95% correlation in the interpretation of growth between the SOC and the APAS workflows combined with the lack of clinically significant growth interpretation differences suggests that the APAS provided a robust platform for interpreting significant growth patterns for urinary workflows.

**TABLE 1:** Growth pattern discrepancies

Data	Value (*n* [%])	95% CI^*a*^ (%)
Total no. of:		
Enrolled specimens	1,519	
Matching growth patterns	1,445 (95.13)	
Discrepancies	74 (4.87)	3.8–6.1
Clinically significant discrepancies	2 (0.13)	0–0.5
Non-clinically significant discrepancies	72 (4.74)	3.7–5.9

a CI, confidence interval.

We based our criteria for determining whether a urine culture was considered positive on the ASM manual criteria (18). We observed that 993 of the 1,519 total cultures (65.4%) were positive, and 526 (34.6%) were negative. There were no discrepancies in growth interpretations between the APAS and SOC workflows for negative cultures. Of the 993 positive urine cultures, 74 (7.45%) were found to have growth discrepancy interpretations between the workflows that required further evaluation of the APAS images. The majority of the positive cultures had only bacterial pathogens identified, but 18 of those cultures also identified yeasts (see Table S1 in the supplemental material). Most of the yeasts found were Candida albicans (12/18; 66.7%), followed closely by Candida glabrata (3/18; 16.7%).

### Identification discrepancies.

**FIG 1:**
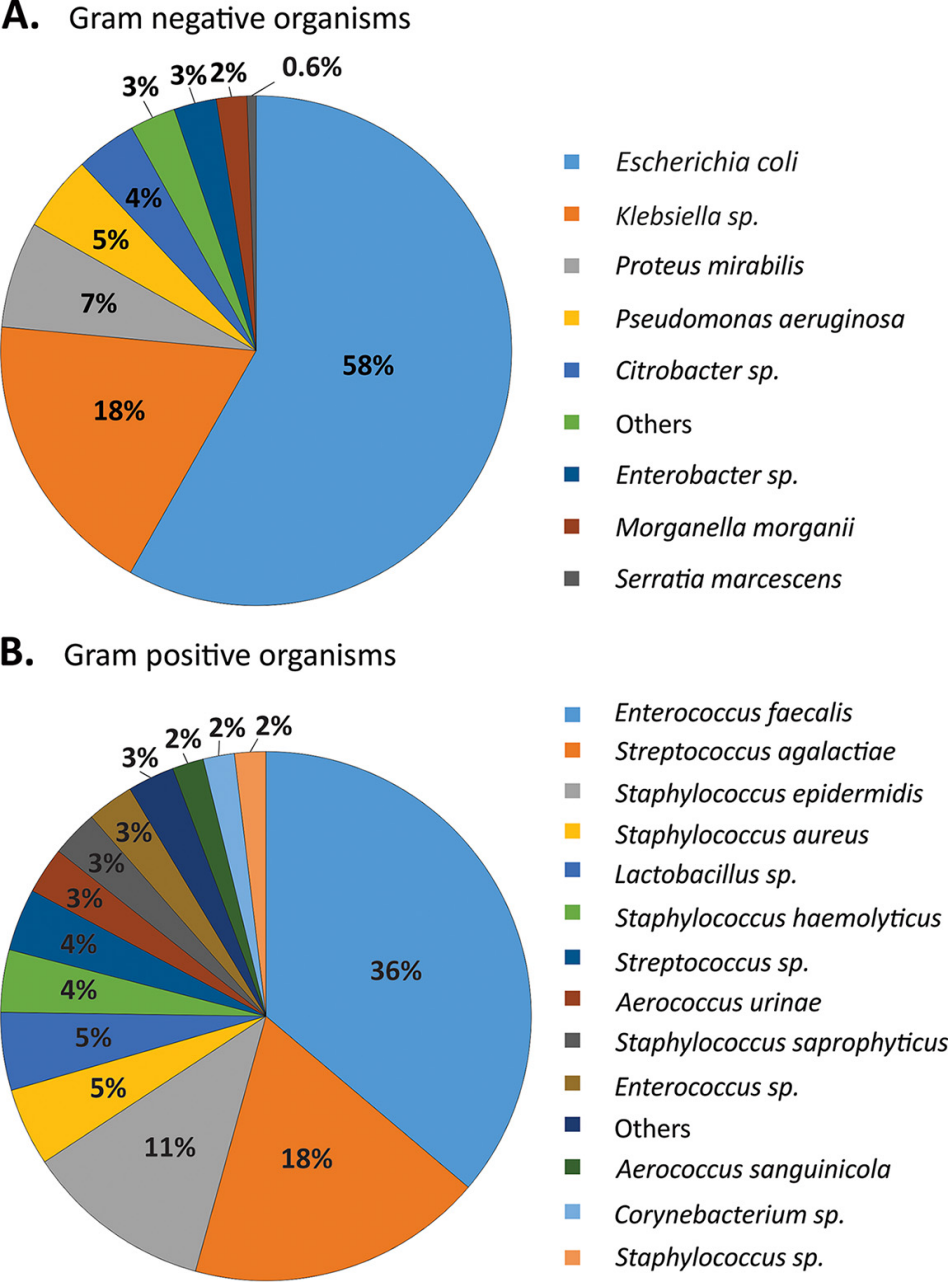
Pie charts representing the relative proportions of Gram-negative (A) and Gram-positive (B) bacteria represented in positive urine cultures in the APAS workflow.

For each culture that we evaluated, we identified putative bacterial and fungal pathogens using the Bruker matrix-assisted laser desorption ionization–time of flight mass spectrometry (MALDI-TOF MS) Microflex instrument. We quantified the numbers of the Gram-positive and Gram-negative bacteria we observed to determine whether we identified similar numbers of these microbes across the APAS and SOC workflows. For the Gram-negative bacteria, using the APAS workflow, we observed significant numbers of E. coli (58%), followed by different species of Klebsiella (18%), Proteus mirabilis (6%), and Pseudomonas aeruginosa (5%) (Fig. 1A). We also identified substantial numbers of Gram-positive bacteria using the APAS workflow, with E. faecalis (36%) as the most prominent, followed by Streptococcus agalactiae, Staphylococcus epidermidis, and Staphylococcus aureus (Fig. 1B). These data indicate that we observed a myriad of different potential pathogens during our evaluation of the APAS workflow.

Of the 933 positive urine cultures that we evaluated, we identified 56 (5.64%) with discrepancies in the identification of pathogens (Table 2). We identified between 0 and 5 of these discrepancies per day of the study, with an average of 2.33 discrepancies out of 63.29 cultures per day. Most of these discrepancies occurred when more potential pathogens were identified using the APAS workflow than the SOC. Of those 56 discrepant culture identifications, 42 (75%) occurred when additional pathogens were found using the APAS workflow, and 14 (25%) occurred when additional pathogens were identified using the SOC workflow (Table 3). While there should be no fundamental difference in the ability of the technologist to identify pathogens on the plates using either workflow, we believe that the flagging of cultures by the APAS resulted in greater scrutiny of each plate for potential pathogens.

**TABLE 2:** Summary of results

Date (mo/day/yr)	No. of:							
	Cultures	Positive cultures	ID discrepancies	Antibiotics reported	AST discrepancies	mEs	MEs	VMEs
7/15/2021	73	39	2	349	6	6	0	0
7/23/2021	82	50	4	330	17	8	8	1
7/27/2021	58	25	2	216	12	3	1	8
7/30/2021	79	41	4	396	13	7	3	3
8/4/2021	82	59	2	513	15	14	1	0
8/10/2021	62	42	1	390	8	4	3	1
8/11/2021	80	49	3	410	5	5	0	0
8/12/2021	80	60	4	491	9	4	5	0
8/16/2021	47	25	0	187	6	4	0	2
8/17/2021	55	42	1	416	9	4	1	4
8/18/2021	66	47	0	303	7	6	0	1
8/24/2021	57	34	4	230	9	3	6	0
8/26/2021	56	39	0	328	4	2	2	0
8/31/2021	54	38	2	252	6	3	2	1
9/2/2021	60	40	5	295	3	3	0	0
9/8/2021	53	33	1	223	13	7	6	0
9/21/2021	57	42	2	323	7	6	1	0
9/23/2021	67	46	2	331	7	5	1	1
9/28/2021	51	34	4	302	8	7	0	1
9/29/2021	78	51	2	422	9	7	1	1
10/5/2021	43	35	2	330	13	12	0	1
10/12/2021	58	38	5	217	5	4	0	1
10/13/2021	85	63	3	512	13	10	0	3
10/14/2021	36	21	1	179	10	3	0	7
Total	1,519	993	56	7,945	214	137	41	36
Percentage		65.37	3.69		2.69	1.72	0.52	0.45
95% CI^*a*^			2.8–4.8		12.4–15.9	7.6–10.5		

a CI, confidence interval.

**TABLE 3:** Identification discrepancies^*a*^

Organism ID	SOC identification	APAS-assisted study identification
APAS_0043	**K. pneumoniae**	MUF^*b*^
APAS_0046	Morganella morganii, **K. pneumoniae**	M. morganii
APAS_0081	E. coli, K. oxytoca	E. coli, K. oxytoca, **K. pneumoniae**
APAS_0083	E. faecalis	E. faecalis, **K. pneumoniae**
APAS_0104	MUF	**Enterococcus faecium**
APAS_0107	**C. glabrata**	MUF
APAS_0166	E. faecalis, **C. albicans**	E. faecalis
APAS_0169	**C. glabrata**	MUF
APAS_0227	E. coli	E. coli, **E. faecalis**
APAS_0230	**Candida lusitaniae**	**C. albicans**
APAS_0241	MUF	**E. coli**, **S. agalactiae**
APAS_0337	MUF	**E. faecalis**
APAS_0362	K. oxytoca, Myroides odoratimimus, **Providencia rettgeri**	K. oxytoca, *M. odoratimimus*, **M. morganii**
APAS_0414	MUF	**C. tropicalis**
APAS_0470	**E. coli**	MUF
APAS_0476	Citrobacter koseri, E. coli	*C. koseri*, E. coli, **E. faecalis**
APAS_0512	MUF	***S. saprophyticus***
APAS_0517	E. coli	E. coli, ***C. koseri***
APAS_0519	MUF	**E. faecalis**, MUF
APAS_0558	E. coli	E. coli, ***C. koseri***
APAS_0562	**P. aeruginosa**	MUF
APAS_0668	K. oxytoca, **E. coli**	K. oxytoca, **Enterobacter** **sp.**
APAS_0765	M. morganii	M. morganii, **K. pneumoniae**
APAS_0778	MUF	**K. pneumoniae**
APAS_0780	S. epidermidis, **Streptococcus gallolyticus**	S. epidermidis
APAS_0782	*S. haemolyticus*	*S. haemolyticus*, ***Candida* sp.**
APAS_0902	MUF	**E. coli**
APAS_0921	**S. aureus**	MUF
APAS_0932	P. aeruginosa	P. aeruginosa, **K. pneumoniae**
APAS_0945	**E. faecalis**	NSG^*c*^
APAS_0960	Citrobacter freundii	C. freundii, **E. faecalis**, **E. coli**
APAS_0964	MUF	**P. mirabilis**
APAS_0983	S. agalactiae	S. agalactiae, **E. faecalis**, **E. coli**
APAS_1016	E. coli, P. mirabilis	E. coli, P. mirabilis, **K. pneumoniae**
APAS_1070	K. pneumoniae	K. pneumoniae, **E. coli**
APAS_1082	E. coli	E. coli, **P. mirabilis**
APAS_1146	E. coli	E. coli, **E. faecalis**
APAS_1152	MUF	**E. coli**
APAS_1170	Candida kefyr	C. kefyr, **C. albicans**
APAS_1186	MUF	**E. faecalis**
APAS_1204	NSG	**K. pneumoniae**
APAS_1206	K. pneumoniae, P. mirabilis	K. pneumoniae, P. mirabilis, **K. oxytoca**
APAS_1276	**Proteus hauseri**	**P. mirabilis**, **E. coli**
APAS_1293	E. coli, **K. oxytoca**	E. coli, **P. aeruginosa**
APAS_1312	MUF	**E. faecalis**
APAS_1313	Klebsiella variicola, **K. pneumoniae**	*K. variicola*
APAS_1341	Klebsiella aerogenes	K. aerogenes, **E. coli**
APAS_1344	Enterobacter cloacae	E. cloacae, **E. faecalis**
APAS_1349	E. coli	E. coli, **K. aerogenes**
APAS_1350	E. faecalis, E. coli	E. faecalis, E. coli, **S. marcescens**
APAS_1352	E. coli, *C. koseri*	E. coli, *C. koseri*, **P. mirabilis**
APAS_1353	*K. variicola*	*K. variicola*, **E. coli**
APAS_1400	MUF	**E. faecalis**
APAS_1445	E. coli, **E. faecalis**	E. coli
APAS_1478	M. morganii	M. morganii, **Citrobacter amalonaticus**
APAS_1488	P. mirabilis	P. mirabilis, **P. aeruginosa**

a Bold indicates microbial isolates identified in only 1 of the 2 workflows.

b MUF, mixed urethral flora.

c NSG, no significant growth.

We identified a number of different cultures with discrepant identifications that spanned many different categories of organisms. For example, we identified Enterococcus faecalis, Staphylococcus saprophyticus, Klebsiella pneumoniae, E. coli, Serratia marcescens, P. aeruginosa, Candida tropicalis, C. albicans, and C. glabrata, among other potential pathogens in these cultures (Table 3). These data suggest that there was not a specific bias in our identification of pathogens using the APAS or SOC workflows, as the discrepancies spanned a large spectrum of microbes.

### Antimicrobial susceptibilities.

In addition to characterizing growth pattern discrepancies and pathogen identifications using the APAS and SOC workflows, we also evaluated ASTs. We performed this analysis in part to evaluate whether we could identify microbes with similar susceptibilities using either workflow. The ASTs were performed using BD Phoenix M50 instruments with AST panels appropriate for each organism evaluated (19). In total, we compared ASTs for 7,945 antibiotic/bacterium combinations. Of note, ASTs were only performed for organisms that were identified at the species level in both the APAS and SOC workflows. We found that there was no significant disagreement in the AST results among the different workflows, with only 214 discrepancies out of the 7,945 total examined (2.69%) (95% CI, 12.4% to 15.9%; Table 2). We identified some discrepancies on each day of the study, with the number of discrepancies ranging between 3 and 17 on any given day.

We next categorized each discrepancy according to its error type, including minor errors (mEs), which were defined as results that were intermediate in one workflow but susceptible or resistant in the other; major errors (MEs), which were defined as resistant by the APAS workflow but susceptible by the SOC; and very major errors (VMEs), which were defined as susceptible by the APAS workflow but resistant by the SOC. We performed ASTs for all potentially pathogenic microbes recovered from the positive urine cultures, which represented 7,945 total ASTs. Of the 214 AST discrepancies we identified (Table 2), 136 (1.71% of the antibiotics reported) were categorized as mEs. This mE number did not meet the threshold for statistical significance (95% CI, 7.6% to 10.5%; Table 2), indicating that the number of mEs was not statistically different between the two workflows. We also identified 41 (0.52%) errors that were categorized as MEs and 36 (0.45%) that were categorized as VMEs. Each of these error types was distributed across time over the length of the study.

While they represent a relatively low proportion of the overall ASTs performed in this study, the MEs and VMEs warranted further investigation. There were 110 different isolates that accounted for the 136 total mEs, indicating that the mEs were spread relatively evenly across the different isolates. We found a different situation for the MEs, where only 17 isolates accounted for the 41 total MEs. Indeed, 8 of the 41 MEs (19.5%) were all present in a single Klebsiella oxytoca isolate. Similarly, the 36 VMEs were accounted for by just 25 isolates. There was a single E. coli isolate that accounted for 6 (16.7%) of the VMEs observed. The relatively limited number of different isolates that accounted for the majority of the MEs and VMEs identified in this study suggests that they may result from the isolation of different strains with heterogenous ASTs.

We also evaluated both the categorical agreement (CA; proportion of isolates with the same categorical interpretation as the SOC method) and essential agreement (EA; proportion of the isolates with the same MIC value as those obtained using the SOC method or within one dilution) in our classification of the AST results and compared the APAS workflow to our SOC. We separated the microbes into Gram positive and Gram negative to discern whether there were observable differences. We identified 105 Gram-positive microbes and performed a total of 341 ASTs against them. We found that all (100%) of the antibiotics had EA, and 337 of the 341 (98.83%) had CA. For the Gram-negative microbes, we identified 519 bacteria from the cultures and performed 7,604 ASTs. We found that 7,527 of the 7,604 organisms (98.99%) had EA, and 7,388 (97.16%) of the 7,604 had CA. The majority of the discrepancies were found in E. coli, followed by Klebsiella and Proteus species (Table S2). While there were some discrepancies in ASTs for both Gram-positive and Gram-negative microbes (Tables 2 and 4), the high percentages of CA and EA indicate that similar results were obtained (Table 4) regardless of whether the APAS or SOC workflows were used.

**TABLE 4:** ASTs for Gram-positive and Gram-negative bacteria

Type of bacteria	No. of organisms	No. of antibiotics	EA^*a*^ (*n* [%])	EA confidence interval (%)	CA^*b*^ (*n* [%])	CA confidence interval (%)
Gram positive	105	341	341 (100)		337 (98.83)	97.0–99.7
Gram negative	519	7,604	7,527 (98.99)	98.7–99.2	7,388 (97.16)	96.8–97.5

a EA, essential agreement.

b CA, categorical agreement.

### Efficiency.

**FIG 2:**
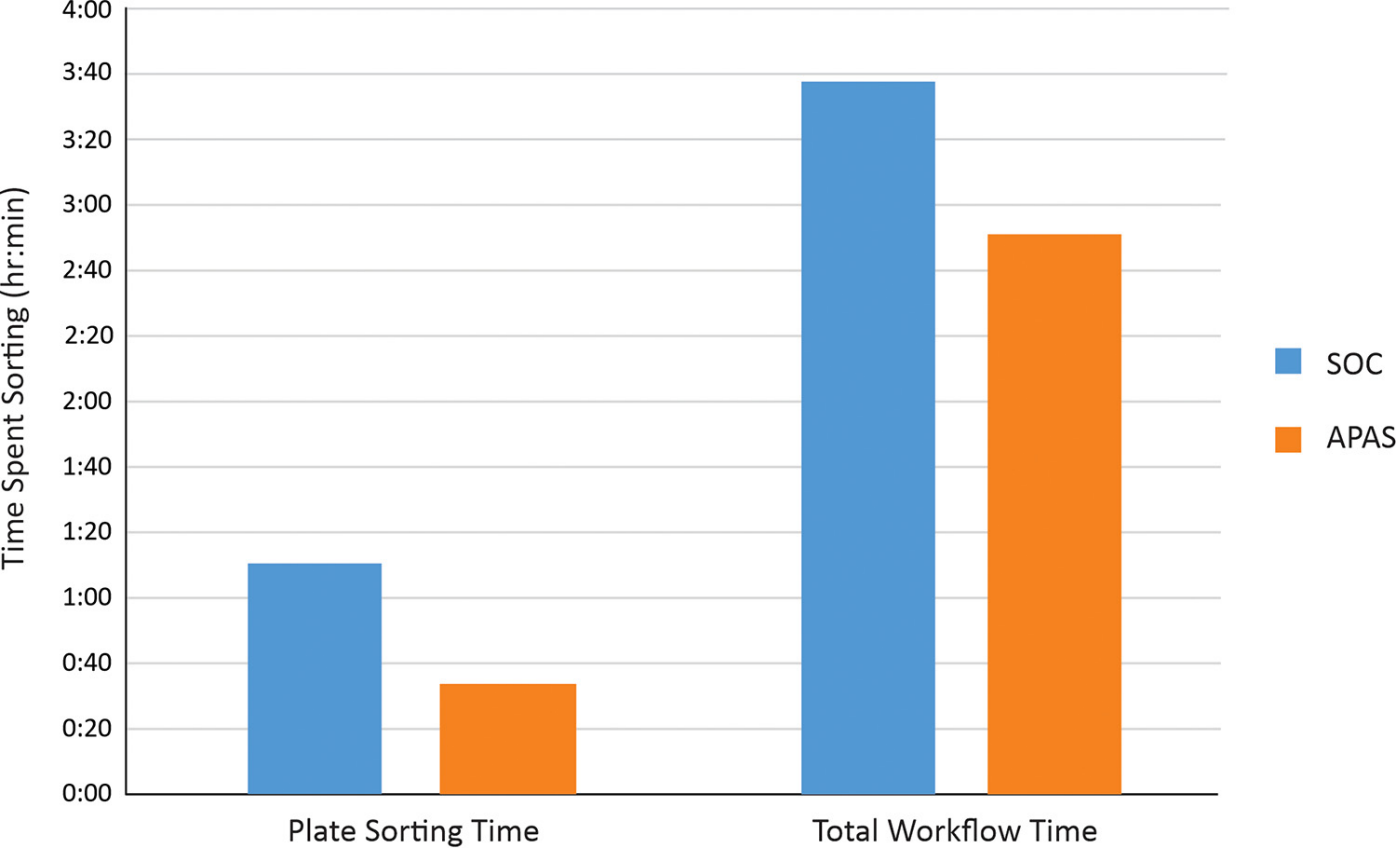
Comparisons of the time required for sorting urine culture plates and for the entire workflow, including identification of the bacteria in urine cultures.

We tracked the time necessary to process the urine specimens using the APAS and SOC workflows. We included the time necessary for sorting the culture plates into specific growth designations for further analysis. In our comparisons of the APAS and SOC sorting workflows, we found that there was a significant difference (*P* < 0.05; Welch’s test) in the times necessary to sort the plates based on growth significance. The APAS-assisted sorting took approximately 34 min to process 64 urine cultures, whereas the SOC took 1 h 11 min to process the same number of cultures (Fig. 2). The overall time to process the specimens from urine sample to identification on the Bruker MALDI-TOF was 3 h 39 min for the SOC and 2 h 47 min for the APAS workflow. This difference of almost 1 h (52 min) to process the urine specimens did not reach statistical significance (*P* = 0.11; Welch’s test). We also further examined the time necessary for sorting plates by comparing the SOC to the APAS workflow on three consecutive days and determining the mean sorting times for plated cultures. The SOC spent an average of 42 s to sort plates, whereas the same process took only 17 s using the APAS workflow. This indicates that the APAS is capable of sorting specimens about 2.5× faster than the SOC (*P* = 0.009) (Table 5). These results indicate that incorporating the APAS into the urine culture workflow could result in significant time savings.

**TABLE 5:** SOC and APAS sorting times

Workflow	Day	Duration (h:min:s)	No. of plates	Time per plate (s)	95% CI (s)^*a*^	*P* value^*a*^
SOC	1	3:30:00	460	46		
	2	2:30:00	364	41		
	3	3:00:00	480	38		
				42^*b*^	14.627–34.706	0.009
APAS	1	00:20:22	72	17		
	2	00:49:22	171	17		
	3	00:20:05	72	17		
				17		

a 95% CI for the mean difference between the SOC and APAS.

b Values in shaded cells represent the mean sorting time per plate (s).

## DISCUSSION

Automation in the clinical laboratory offers a means of expediting culture results and reducing reliance on manual labor (11). We used the APAS Independence instrument, which provides an automated means of visualizing, interpreting, and sorting urine culture plates, to analyze whether it could expedite urine culture results, reduce manual labor, and provide high-quality results. We compared APAS workflow results with those of our SOC by performing two separate workflows on the same urine specimens. We examined the growth patterns for potential discrepancies, microbial identifications, and ASTs to decipher whether similar results could be obtained using either workflow. Our results indicate that we can expedite the results of urine cultures using this system (Table 5), while largely identifying the pathogens responsible for UTIs and determining their ASTs, with no significant differences in the AST results compared (Tables 2 and 4) to the SOC workflow. Most of the differences in interpretation between the APAS and the SOC involved additional microbes being identified using the APAS workflow (Table 3).

A number of platforms are available to help expedite the results of urine cultures through the automation of many of the manual steps involved in urine culture workflow. For example, the WASP instrument available from Copan (9) and the InoqulA sample processor from Becton, Dickinson and Company (BD) allow for the automated plating of urine specimens to reduce manual labor. The Copan WASP instrument was utilized in this study as part of both the SOC and the APAS workflows (10). The use of digital images for interpretation of urine plates has been demonstrated using Copan WASPLab segregation software (20) and BD Kiestra (21). However, these current platforms do require the user to inspect and verify the segregated results. Each of these platforms can expedite results and reduce manual labor, but they do not result in nearly complete automation of urine culture processing. The Copan WASPLab and BD Kiestra system require significant changes to laboratories to take full advantage of the automation (11). The major benefit of the APAS system is its ability to expedite urine culture interpretation while fitting into a standard laboratory workflow, rather than necessitating an overhaul of laboratory workflows (12). When the system is fully implemented, it can report no-growth urine cultures to laboratory information systems without having to manually interpret or further manipulate those plates, which is a key differentiator from both the Copan and BD systems, which require manual confirmation on no-growth plates for release. Not requiring manual verification of the results is advantageous because it cuts down the time needed for patients to receive a final report. This key difference has resulted in the classification of the APAS Independence as a class II medical device in the United States, and it is the only such classified device (4). While our laboratory urine culture volumes only resulted in a savings of approximately 1 h of hands-on time, greater urine culture volumes likely would have resulted in more substantial time and labor savings. Laboratories that encounter lower urine culture positivity rates than were observed in this study could experience an even greater increase in workflow efficiency by incorporating the APAS Independence instrument, since a majority of the plates would no longer require manual observation.

We evaluated discrepancies in the identification of urinary pathogens between our SOC workflow and the APAS. While each of the specimens was plated twice (once for the SOC and again for the APAS), we noted some differences in the ability to identify pathogens between the workflows. The proportion of identification discrepancies was relatively low (3.69%), but there were some significant differences that could have resulted in different treatment decisions. We noted that approximately 75% of those cultures in which additional pathogens were identified occurred in the APAS rather than the SOC workflow. We believe that the APAS workflow difference resulted in us identifying these additional isolates. Because the instrument flagged these cultures as abnormal and requiring further attention, the technologists evaluating them likely scrutinized these cultures more thoroughly, resulting in the additional isolates identified. We realize that some discrepancies could be caused by plating the same specimens twice, but we were not able to determine definitively whether the APAS or SOC workflows resulted in more accurate results. Regardless, because the level of discrepancies was low, incorporating the APAS into laboratory workflows is unlikely to result in significant differences in SOC reporting.

One of the most salient features of microbiology is determining antibiotic susceptibility profiles for pathogens in urine and other body sources. This process generally involves the subculture of a single colony from the specimen to ensure its purity before performing an AST. In this study, the ASTs were performed on the BD Phoenix instrument using the appropriate testing panels. While the vast majority of the ASTs performed using the APAS and SOC workflows were in agreement (Table 4), about 2.69% were discrepant. We noted the presence of mEs, MEs, and VMEs in our data set. While the mEs were unlikely to significantly impact the care of the patients, the MEs and VMEs could have altered antibiotic prescriptions and potentially patient outcomes. With such a large number of urine cultures examined, we expected to identify a few MEs and VMEs, but we did not expect to identify as many as 41 MEs and 36 VMEs. Many of these errors were present in the same isolates, with the 41 MEs occurring in only 17 different isolates and the 36 VMEs occurring in only 25 different isolates. We found that several isolates had high numbers of MEs and VMEs. These data suggest that we likely identified different isolates of the same species that had differing antimicrobial susceptibilities. We did not sequence the isolates to determine whether they represent different strains of the same species, but the high number of antimicrobial susceptibilities suggests that they were different. There are substantial numbers of reports of heterogenous isolates in urine specimens resulting in differing antibiotic susceptibilities (22).

### Conclusion.

Using the APAS instrument could reduce the hands-on-time required for processing urinary cultures, while providing a similar set of results as those produced by our SOC. The APAS workflow still involves manual steps in processing and incubating samples compared to nearly fully automated workflows. Thus, the reduction in hands-on-time is not as great as it is in total laboratory automation systems. However, the benefits of the APAS system are that it can be incorporated into the workflows of most laboratories with only minor adjustments, and the system can be installed and start running within only a few days. While the instrument can reduce the hands-on time to result in full-time equivalent (FTE) hour savings, the FTE savings are likely to be volume dependent, as one might expect. An obvious benefit to the APAS system is that negative cultures can be automatically reported in real time without user intervention, resulting in additional clinical benefits through a decreased time to report. Studies into the latter would be needed to determine if this is a possibility and what additional benefits could be achieved. The APAS instrument is also capable of supporting the reading and interpretation of other cultures in the lab, such as evaluating methicillin-resistant S. aureus (MRSA) CHROMagar plates (4), where a positive percent agreement of 100% and negative predictive value of 100% were achieved. This could add significantly to its utilization in reducing FTEs in the laboratory, as well as ensuring that the quality of results released was in line with laboratory procedures.

## MATERIALS AND METHODS

### Human subject protections.

This study was reviewed by the UCSD Human Research Protections Program and certified as exempt from institutional review board (IRB) review under 45 CFR 46.104(d), category 4.

### Standard of care workflow.

The urine cultures were inoculated separately via the Copan WASP system using a 1-μL loop onto Sheep’s blood agar and MacConkey agar plates and then incubated at 37 ± 2°C for 2 days. We evaluated urine from a number of different patient sources, including clean catch samples and catheter-, nephrostomy-, and suprapubic catheter-collected specimens. The cultures were examined after 24 h by a clinical laboratory scientist (CLS), and final readings of all plates regardless of growth were made after 48 h of incubation. For enumeration as per the ASM manual criteria, one colony equates to 1,000 CFU/mL, and growth at ≥10^5^ CFU/mL was considered to represent a positive culture, which was then further evaluated for the quantity and morphology of the colonies (18). Streptococcus agalactiae was only reported for women in childbearing years (14 to 50 years old) if present in the amount of ≥10^4^ CFU/mL, and antimicrobial susceptibility testing was only performed on this organism upon physician request. This diagnostic protocol is in accordance with the Centers for Disease Control and Prevention (CDC) recommendation to screen women who are between 35 and 37 weeks of gestation for S. agalactiae (23).

### APAS Independence workflow.

The urine cultures, originating from samples of all different urine collection types, for the APAS Independence workflow were inoculated using the Copan WASP system and incubated according to the SOC protocol. The resulting plates were loaded onto the APAS Independence machine’s input section, and a session was initiated to run. The instrument took images of the plates from a top and bottom view, performed an interpretation of the images, and sorted the plates into the output section. It interpreted no growth as “no growth,” growth of ≤10^3^ CFU/mL as “doubtful,” growth of ≥10^4^ CFU/mL with a predominant/pure organism as “probable,” and mixed growth of ≥10^4^ CFU/mL as requiring review. The throughput of the instrument is approximately 200 plate reads per hour, with a total loading capacity of 240 plates. After the plates were sorted, they were reviewed a second time by a staff technician to confirm growth patterns. The urine culture plates, regardless of growth, were reincubated for an additional 24 h for further analysis, sorted through the APAS instrument, and interpreted again at 48 h. If discrepancies in growth were noted compared to the SOC workflow after the 24- or 48-h incubation time, the images taken by the APAS were given an additional manual evaluation. Cultures with significant organism growth were then evaluated according to the SOC protocol.

### Bacterial identification.

After the plates were sorted, bacterial and yeast identification was performed for colonies that demonstrated significant growth using the Bruker MALDI-TOF MS Microflex instrument. Each colony with significant growth that represented a different morphotype was picked from the culture plate using a toothpick and applied in duplicate (or greater) to the Bruker MALDI-TOF MS target to dry. Then, 1 μL 70% formic acid was added to each spot and allowed to dry for 5 min or longer. Following this, 1 μL Bruker matrix HCCA (α-cyano-4-hydroxycinnamic acid) was added and allowed to dry for an additional 5 min or longer. Finally, each target spot was compared to the Compass Explorer version 4.1.100.0 research use only database for identification. The organism identification results were considered valid when a minimum of 2 separate spots had confidence scores of ≥2.0 for the same microbiological identification.

To compare sorting times between the SOC and the APAS workflows, we tracked the read times on three separate days. For the SOC, two microbiologists were assigned to read urine culture plates each day. The first microbiologist screened the urine culture plates and sorted the plates based on growth. The second microbiologist read and analyzed the prescreened plates. Their read times for plate interpretations were recorded. This measurement included the time it took them to remove the urine culture plates from the incubator, de-rack and sort them, and interpret the growth. The read time ended when the urine culture plates were ready for ID and AST preparation. The total number of urine culture plates were counted for each day of observation. We calculated the mean read time per plate for all 3 days. Similarly, we calculated the average time for APAS-assisted reads per plate by looking at three independent sessions, from removal of the plates from the incubator until the plates were ready for ID and AST preparation.

### AST analysis.

Once a bacterial ID was confirmed, AST was performed on the isolates using the BD Phoenix M50 system (Becton, Dickinson and Company). Each bacterium identified was used to prepare a 0.5 McFarland standard in BD Phoenix ID broth (Becton, Dickinson and Company). Depending on the bacterium’s identity, it was tested on positive minimal inhibitory concentration (PMIC), negative minimal inhibitory concentration (NMIC), or streptococcus minimal inhibitory concentration (SMIC) panels. MIC and AST interpretations were available after approximately 24 h and were recorded to calculate the categorical agreement (CA; proportion of APAS AST interpretation with the same categorical SIR [susceptible, intermediate, resistant] interpretation as the SOC method) and essential agreement (EA; proportion of APAS MIC values within one dilution of those obtained using the SOC method). AST differences were also categorized into 3 different error types: minor errors (mEs), major errors (MEs), and very major errors (VMEs). mEs were defined as results that were susceptible or resistant in one system but intermediate in the other. MEs were defined as susceptible by the SOC results but resistant by the APAS results. Finally, VMEs were defined as resistant by the SOC results but susceptible by the APAS results.

### Statistics.

To determine whether differences in the growth patterns observed between the APAS and the SOC workflows were statistically significant, we compared the proportion of discrepancies in growth patterns using exact confidence intervals for binomial proportions (24). The exact approach is necessary to deal with the relatively low proportion of discrepancy, as asymptotic methods will yield inaccurate results (24). The same approach was used for the comparison of mEs, MEs, VMEs, CA, and EA for the AST results, which necessitates the exact method, as the observed proportions were too close to 1 (24). For comparisons of sorting times, we performed the *t* test to compare the mean differences in sorting times between the workflows, assuming unequal variances. All these comparisons were performed using R software (25).
