# Supplementary material for: Comparison of the APAS Independence Automated Plate Reader System with the Manual Standard of Care for Processing Urine Culture Specimens
Source: Microbiol Spectr. 2022 Aug 16;10(5):e01442-22. doi: 10.1128/spectrum.01442-22 (PMC9603219; doi:10.1128/spectrum.01442-22)
Supplement: Supplemental file 1 — Supplemental material. Download spectrum.01442-22-s0001.pdf, PDF file, 0.4 MB [file spectrum.01442-22-s0001.pdf]

Table S1: Yeasts identified in this study

Identification	Number (Percentage of positive cultures)
C. albicans	12/993 (1.21%)
C. glabrata	3/993 (0.30%)
C. lusitaniae	1/993 (0.10%)
C. kefyr	1/993 (0.10%)
C. krusei	1/993 (0.10%)
**Total**	**18/993 (1.81%)**

Table S2: Antibiotic Susceptibility Errors

Specimen Number	Isolate	Antibiotic	SOC		Study		Type of Error
			MIC	Interpretation	MIC	Interpretation	
APAS_0008	*E. coli*	Tobramycin	8	Intermediate	4	Susceptible	mE
APAS_0017	*P. mirabilis*	Amikacin	32	Intermediate	16	Susceptible	mE
		Tobramycin	8	Intermediate	>8	Resistant	mE
APAS_0018	*E. coli*	Ampicillin-Sulbactam	>16/8	Resistant	16/8	Intermediate	mE
APAS_0031	*P. mirabilis*	Cefazolin	8	Susceptible	16	Intermediate	mE
APAS_0046	*M. morganii*	Ampicillin-Sulbactam	16/8	Intermediate	>16/8	Resistant	mE
**APAS_0081**	***K. oxytoca***	**Ceftazidime**	**<=2**	**Susceptible**	**>16**	**Resistant**	**ME**
		**Ciprofloxacin**	**<=2**	**Susceptible**	**>2**	**Resistant**	**ME**
		**Ceftriaxone**	**<=1**	**Susceptible**	**>32**	**Resistant**	**ME**
		**Cefazolin**	**8**	**Susceptible**	**<16**	**Resistant**	**ME**
		**Cefepime**	**<=1**	**Susceptible**	**16**	**Resistant**	**ME**
		**Nitrofurantoin**	**<=16**	**Susceptible**	**>64**	**Resistant**	**ME**
		**Gentamicin**	**<=2**	**Susceptible**	**>8**	**Resistant**	**ME**
		**Tobramycin**	**<=2**	**Susceptible**	**>8**	**Resistant**	**ME**
		Ampicillin-Sulbactam	16/8	Intermediate	>16/8	Resistant	mE
APAS_0088	*E. coli*	Cefepime	8	Intermediate	>16	Resistant	mE
		Tobramycin	8	Intermediate	4	Susceptible	mE
APAS_0116	*K. oxytoca*	Ampicillin-Sulbactam	16/8	Intermediate	8/4	Susceptible	mE
APAS_0140	*K. pneumoniae*	Ceftazidime	>16	Resistant	16	Intermediate	mE
		Piperacillin-Tazobactam	16/4	Susceptible	32/4	Intermediate	mE
**APAS_0151**	***C. koseri***	**Nitrofurantoin**	**>64**	**Resistant**	**<=16**	**Susceptible**	**VME**
APAS_0153	*E. coli*	Cefepime	>16	Resistant	8	Intermediate	mE
APAS_0155	*P. mirabilis*	Ciprofloxacin	1	Resistant	0.5	Intermediate	mE
**APAS_0161**	***E. coli***	**Cefazolin**	**8**	**Susceptible**	**>16**	**Resistant**	**ME**
**APAS_0173**	***E. coli***	**Ampicillin**	**>16**	**Resistant**	**<=4**	**Susceptible**	**VME**
		**Ciprofloxacin**	**>2**	**Resistant**	**<=0.25**	**Susceptible**	**VME**
		**Ceftriaxone**	**>32**	**Resistant**	**<=1**	**Susceptible**	**VME**
		**Cefazolin**	**>16**	**Resistant**	**4**	**Susceptible**	**VME**
		Cefepime	4	Intermediate	<=1	Susceptible	mE
		**Gentamicin**	**>8**	**Resistant**	**<=2**	**Susceptible**	**VME**
		**Tobramycin**	**>8**	**Resistant**	**<=2**	**Susceptible**	**VME**
		Ampicillin-Sulbactam	16/8	Intermediate	<=1/0.5	Susceptible	mE
**APAS_0189**	***E. coli* (2 strains)**	**Trimethoprim-Sulfamethoxazole**	**>2/38**	**Resistant**	**<=0.5/9.5**	**Susceptible**	**VME**

**APAS_0200**	***K. pneumoniae* (2 strains)**	**Nitrofurantoin**	**>64**	**Resistant**	**32**	**Susceptible**	**VME**
APAS_0207	*E. coli*	Cefepime	8	Intermediate	>16	Resistant	mE
APAS_0226	*K. oxytoca*	Cefazolin	4	Susceptible	16	Intermediate	mE
**APAS_0227**	***E. coli***	**Ceftazidime**	**4**	**Susceptible**	**>16**	**Resistant**	**ME**
		Cefepime	8	Intermediate	>16	Resistant	mE
		**Meropenem**	**<=0.5**	**Susceptible**	**4**	**Resistant**	**ME**
		**Tobramycin**	**>=2**	**Susceptible**	**>8**	**Resistant**	**ME**
**APAS_0253**	***E. coli***	**Nitrofurantoin**	**>64**	**Resistant**	**32**	**Susceptible**	**VME**
**APAS_0280**	***E. coli***	**Ampicillin**	**>16**	**Resistant**	**<=4**	**Susceptible**	**VME**
		Cefazolin	16	Intermediate	<=1	Susceptible	mE
		**Ampicillin-Sulbactam**	**>16/8**	**Resistant**	**2/1**	**Susceptible**	**VME**
APAS_0288	*E. coli*	Ertapenem	<=0.25	Susceptible	1	Intermediate	mE
		Meropenem	<=0.5	Susceptible	2	Intermediate	mE
APAS_0292	*E. coli*	Ceftazidime	16	Intermediate	8	Susceptible	mE
		Cefepime	8	Intermediate	>16	Resistant	mE
APAS_0294	*K. pneumoniae*	Cefepime	8	Intermediate	>16	Resistant	mE
		Tobramycin	>8	Resistant	8	Intermediate	mE
APAS_0303	*P. mirabilis*	Ampicillin	>16	Resistant	16	Intermediate	mE
APAS_0305	*E. coli*	Cefepime	8	Intermediate	16	Resistant	mE
APAS_0306	*K. pneumoniae* (Strain 2)	Tobramycin	>8	Resistant	8	Intermediate	mE
		Piperacillin-Tazobactam	>64/4	Resistant	32/4	Intermediate	mE
APAS_0332	*K. pneumoniae*	Nitrofurantoin	32	Susceptible	64	Intermediate	mE
APAS_0362	*M. odoratimimus*	Aztreonam	<=2	Susceptible	16	Intermediate	mE
		Ciprofloxacin	2	Intermediate	>2	Resistant	mE
		Tetracycline	<=2	Susceptible	8	Intermediate	mE
APAS_0365	*K. aerogenes*	Nitrofurantoin	64	Intermediate	32	Susceptible	mE
APAS_0373	*K. pneumoniae*	Cefepime	8	Intermediate	>16	Resistant	mE
		Tobramycin	4	Susceptible	8	Intermediate	mE
**APAS_0374**	***M. morganii***	**Ciprofloxacin**	**<=0.25**	**Susceptible**	**>2**	**Resistant**	**ME**
		Tobramycin	8	Intermediate	4	Susceptible	mE
APAS_0382	*P. mirabilis*	Ampicillin-Sulbactam	8/4	Susceptible	16/8	Intermediate	mE
APAS_0386	*S. aureus*	Cefazolin	16	Intermediate	8	Susceptible	mE
**APAS_0389**	***P. mirabilis***	**Nitrofurantoin**	**>64**	**Resistant**	**<=16**	**Susceptible**	**VME**
**APAS_0409**	***M. morganii***	**Ceftriaxone**	**<=1**	**Susceptible**	**8**	**Resistant**	**ME**
		**Ertapenem**	**<=0.25**	**Susceptible**	**>1**	**Resistant**	**ME**
		**Meropenem**	**<=0.5**	**Susceptible**	**>8**	**Resistant**	**ME**
		Ampicillin-Sulbactam	16/8	Intermediate	>16/8	Resistant	mE
APAS_0427	*E. coli*	Tobramycin	8	Intermediate	4	Susceptible	mE

APAS_0440	*K. pneumoniae*	Piperacillin-Tazobactam	64/4	Intermediate	>64/4	Resistant	mE
APAS_0484	*P. mirabilis*	Cefazolin	8	Susceptible	16	Intermediate	mE
APAS_0486	*K. oxytoca*	Ampicillin-Sulbactam	4/8	Susceptible	16/8	Intermediate	mE
APAS_0510	*E. coli*	Piperacillin-Tazobactam	32/4	Intermediate	8/4	Susceptible	mE
APAS_0513	*E. coli*	Piperacillin-Tazobactam	32/4	Intermediate	>644	Resistant	mE
**APAS_0517**	***E. coli***	**Ertapenem**	**<=0.25**	**Susceptible**	**>1**	**Resistant**	**ME**
		Cefepime	>16	Resistant	4	Intermediate	mE
		Tobramycin	>8	Resistant	8	Intermediate	mE
		Ampicillin-Sulbactam	>16/8	Resistant	16/8	Intermediate	mE
**APAS_0536**	***P. aeruginosa***	Aztreonam	8	Susceptible	16	Intermediate	mE
		**Ceftazidime**	**4**	**Susceptible**	**>16**	**Resistant**	**ME**
		**Cefepime**	**4**	**Susceptible**	**16**	**Resistant**	**ME**
		**Meropenem**	**2**	**Susceptible**	**8**	**Resistant**	**ME**
		**Piperacillin-Tazobactam**	**16/4**	**Susceptible**	**>64/4**	**Resistant**	**ME**
**APAS_0606**	*E. coli*	Ampicillin-Sulbactam	>16/8	Resistant	16/8	Intermediate	mE
	C. koseri	**Ampicillin-Sulbactam**	**>16/8**	**Resistant**	**4/2**	**Susceptible**	**VME**
APAS_0624	*S. aureus*	Tetracycline	8	Intermediate	4	Susceptible	mE
APAS_0627	*E. coli*	Ampicillin-Sulbactam	16/8	Intermediate	8/4	Susceptible	mE
**APAS_0634**	***E. coli***	Ciprofloxacin	0.5	Intermediate	<=0.25	Susceptible	mE
		**Trimethoprim-Sulfamethoxazole**	**>2/38**	**Resistant**	**1/19**	**Susceptible**	**VME**
APAS_0645	*E. coli*	Ceftazidime	16	Intermediate	>16	Resistant	mE
**APAS_0646**	***E. coli***	**Trimethoprim-Sulfamethoxazole**	**>2/38**	**Resistant**	**2/38**	**Susceptible**	**VME**
APAS_0647	*E. coli*	Nitrofurantoin	64	Intermediate	32	Susceptible	mE
**APAS_0672**	***C. freundii***	**Nitrofurantoin**	**>64**	**Resistant**	**<=16**	**Susceptible**	**VME**
		**Tetracycline**	**>8**	**Resistant**	**<=2**	**Susceptible**	**VME**
	P. mirabilis	Ampicillin-Sulbactam	16/8	Intermediate	2/1	Susceptible	mE
**APAS_0676**	***P. mirabilis***	**Ampicillin-Sulbactam**	**>16/8**	**Resistant**	**4/2**	**Susceptible**	**VME**
APAS_0692	*E. coli*	Ampicillin-Sulbactam	>16/8	Resistant	16/8	Intermediate	mE
**APAS_0695**	***E. coli***	**Trimethoprim-Sulfamethoxazole**	**<=0.5/9.5**	**Susceptible**	**>2/38**	**Resistant**	**ME**
APAS_0706	*P. mirabilis*	Cefazolin	16	Intermediate	>16	Resistant	mE
**APAS_0741**	***P. mirabilis***	Ciprofloxacin	<=0.25	Susceptible	0.5	Intermediate	mE
		**Cefazolin**	**>16**	**Resistant**	**8**	**Susceptible**	**VME**
	P. aeruginosa	Aztreonam	16	Intermediate	8	Susceptible	mE

		Ciprofloxacin	1	Intermediate	2	Resistant	mE
APAS_0743	*P. mirabilis*	Gentamicin	8	Intermediate	4	Susceptible	mE
APAS_0759	*K. pneumoniae*	Nitrofurantoin	64	Intermediate	32	Susceptible	mE
APAS_0765	*M. morganii*	Ceftazidime	8	Susceptible	16	Intermediate	mE
**APAS_0787**	***E. coli***	**Ceftriaxone**	**<=1**	**Susceptible**	**16**	**Resistant**	**ME**
		**Ertapenem**	**<=0.25**	**Susceptible**	**>1**	**Resistant**	**ME**
		**Meropenem**	**<=0.5**	**Susceptible**	**>8**	**Resistant**	**ME**
		**Ampicillin-Sulbactam**	**4/2**	**Susceptible**	**>16/8**	**Resistant**	**ME**
**APAS_0798**	***P. mirabilis***	**Ampicillin**	**<=4**	**Susceptible**	**>16**	**Resistant**	**ME**
		**Ampicillin-Sulbactam**	**2/1**	**Susceptible**	**>16/8**	**Resistant**	**ME**
APAS_0800	*C. koseri*	Nitrofurantoin	32	Susceptible	64	Intermediate	mE
APAS_0818	*E. coli*	Ampicillin-Sulbactam	16/8	Intermediate	8/4	Susceptible	mE
**APAS_0845**	***K. pneumoniae***	**Ertapenem**	**<=0.25**	**Susceptible**	**>1**	**Resistant**	**ME**
		**Meropenem**	**<=0.5**	**Susceptible**	**4**	**Resistant**	**ME**
APAS_0861	*C. freundii*	Ampicillin	8	Susceptible	16	Intermediate	mE
APAS_0875	*E. coli*	Tobramycin	8	Intermediate	>8	Resistant	mE
APAS_0885	*S. saprophyticus*	Cefazolin	16	Intermediate	>16	Resistant	mE
**APAS_0889**	***K. pneumoniae***	**Nitrofurantoin**	**<=16**	**Susceptible**	**>64**	**Resistant**	**ME**
**APAS_0894**	***E. coli***	**Piperacillin-Tazobactam**	**>64/4**	**Resistant**	**4/4**	**Susceptible**	**VME**
APAS_0911	*E. coli*	Cefepime	16	Resistant	8	Intermediate	mE
**APAS_0913**	***E. coli***	**Ertapenem**	**<=0.25**	**Susceptible**	**>1**	**Resistant**	**ME**
APAS_0924	*K. pneumoniae*	Nitrofurantoin	64	Intermediate	32	Susceptible	mE
APAS_0965	*E. coli*	Cefepime	8	Intermediate	16	Resistant	mE
APAS_0967	*K. aerogenes*	Piperacillin-Tazobactam	>64/4	Resistant	64/4	Intermediate	mE
APAS_0975	*E. coli*	Cefazolin	16	Intermediate	8	Susceptible	mE
APAS_0998	*C. koseri*	Nitrofurantoin	64	Intermediate	32	Susceptible	mE
**APAS_1017**	*K. aerogenes*	Nitrofurantoin	64	Intermediate	32	Susceptible	mE
	E. coli	Ceftazidime	<=2	Susceptible	16	Intermediate	mE
		**Ciprofloxacin**	**<=0.25**	**Susceptible**	**>2**	**Resistant**	**ME**
		**Ceftriaxone**	**<=1**	**Susceptible**	**>2**	**Resistant**	**ME**
		**Cefepime**	**<=1**	**Susceptible**	**>16**	**Resistant**	**ME**
		Nitrofurantoin	64	Intermediate	<=16	Susceptible	mE
		**Gentamicin**	**<=2**	**Susceptible**	**>8**	**Resistant**	**ME**
		**Tobramycin**	**<=2**	**Susceptible**	**>8**	**Resistant**	**ME**
		Ampicillin-Sulbactam	16/8	Intermediate	>16/8	Resistant	mE
		**Trimethoprim-Sulfamethoxazole**	**<=0.5/9.5**	**Susceptible**	**>2/38**	**Resistant**	**ME**
APAS_1036	*K. pneumoniae*	Nitrofurantoin	64	Intermediate	32	Susceptible	mE

	P. mirabilis	Cefazolin	16	Intermediate	8	Susceptible	mE
APAS_1049	*E. coli*	Ampicillin-Sulbactam	16/8	Intermediate	>16/8	Resistant	mE
APAS_1054	*E. coli*	Piperacillin-Tazobactam	32/4	Intermediate	16/4	Susceptible	mE
APAS_1070	*K. pneumoniae*	Nitrofurantoin	32	Susceptible	64	Intermediate	mE
APAS_1075	*P. mirabilis*	Gentamicin	4	Susceptible	8	Intermediate	mE
**APAS_1100**	***P. rettgeri***	**Ampicillin**	**>16**	**Resistant**	**<=4**	**Susceptible**	**ME**
APAS_1076	*E. coli*	Tobramycin	8	Intermediate	4	Susceptible	mE
APAS_1082	*E. coli*	Ampicillin-Sulbactam	>16/8	Resistant	16/8	Intermediate	mE
**APAS_1143**	***K. pneumoniae* (strain 1)**	**Ampicillin-Sulbactam**	**18/8**	**Resistant**	**8/4**	**Susceptible**	**VME**
	K. pneumoniae (strain 2)	Nitrofurantoin	>64	Resistant	64	Intermediate	mE
APAS_1149	*A. pitti*	Ceftriaxone	16	Intermediate	8	Susceptible	mE
APAS_1145	*E. coli* (Strain 2)	Tobramycin	4	Susceptible	8	Intermediate	mE
APAS_1161	*K. pneumoniae*	Ciprofloxacin	0.5	Intermediate	<=0.25	Susceptible	mE
		Nitrofurantoin	>64	Resistant	64	Intermediate	mE
**APAS_1162**	***E. coli***	**Cefazolin**	**8**	**Susceptible**	**>16**	**Resistant**	**ME**
APAS_1206	*K. pneumoniae*	Nitrofurantoin	64	Intermediate	32	Susceptible	mE
	P. mirabilis	Cefazolin	8	Susceptible	16	Intermediate	mE
		Nitrofurantoin	>64	Resistant	64	Intermediate	mE
APAS_1209	*E. coli*	Tobramycin	8	Intermediate	>8	Resistant	mE
APAS_1211	*K. pneumoniae*	Ceftazidime	8	Susceptible	16	Intermediate	mE
APAS_1213	*E. coli*	Cefepime	4	Intermediate	>16	Resistant	mE
		Tobramycin	8	Intermediate	>8	Resistant	mE
**APAS_1215**	***K. pneumoniae***	**Nitrofurantoin**	**>64**	**Resistant**	**32**	**Susceptible**	**VME**
APAS_1244	*S. aureus*	Tetracycline	4	Susceptible	6	Intermediate	mE
APAS_1278	*C. koseri*	Nitrofurantoin	>64	Resistant	64	Intermediate	mE
APAS_1279	*K. pneumoniae*	Nitrofurantoin	64	Intermediate	32	Susceptible	mE
**APAS_1282**	***E. coli***	Ampicillin	<=4	Susceptible	16	Intermediate	mE
		**Ciprofloxacin**	**>2**	**Resistant**	**<=0.25**	**Susceptible**	**VME**
APAS_1283	*E. coli*	Cefepime	4	Intermediate	<=1	Susceptible	mE
APAS_1289	*K. aerogenes*	Nitrofurantoin	>64	Resistant	64	Intermediate	mE
APAS_1291	*P. mirabilis*	Gentamicin	4	Susceptible	8	Intermediate	mE
**APAS_1292**	***K. oxytoca***	**Cefazolin**	**8**	**Susceptible**	**>16**	**Resistant**	**ME**
APAS_1308	*K. pneumoniae* (Strain 1)	Ceftazidime	16	Intermediate	>16	Resistant	mE
		Tobramycin	>8	Resistant	8	Intermediate	mE
	K. pneumoniae (Strain 2)	Nitrofurantoin	>64	Resistant	64	Intermediate	mE
APAS_1309	*E. coli*	Ceftazidime	8	Susceptible	16	Intermediate	mE

APAS_1310	*E. cloacae*	Tobramycin	>8	Resistant	8	Intermediate	mE
**APAS_1313**	***K. variicola***	Ceftazidime	16	Intermediate	<=2	Susceptible	mE
		**Ceftriaxone**	**8**	**Resistant**	**<=1**	**Susceptible**	**VME**
		Cefazolin	>16	Resistant	16	Intermediate	mE
APAS_1314	*E. coli*	Tobramycin	8	Intermediate	>8	Resistant	mE
APAS_1319	*E. coli*	Ceftazidime	8	Susceptible	16	Intermediate	mE
APAS_1328	*K. aerogenes*	Ceftazidime	16	Intermediate	>16	Resistant	mE
		Nitrofurantoin	64	Intermediate	32	Susceptible	mE
APAS_1344	*E. cloacae*	Nitrofurantoin	>64	Resistant	64	Intermediate	mE
APAS_1348	*K. oxytoca*	Nitrofurantoin	64	Intermediate	>64	Resistant	mE
**APAS_1352**	*C. koseri*	Nitrofurantoin	>64	Resistant	64	Intermediate	mE
	E. coli	**Nitrofurantoin**	**>64**	**Resistant**	**<=16**	**Susceptible**	**VME**
APAS_1341	*K. aerogenes*	Nitrofurantoin	64	Intermediate	64	Susceptible	mE
APAS_1453	*K. pneumoniae*	Nitrofurantoin	>64	Resistant	64	Intermediate	mE
**APAS_1461**	***E. coli***	**Trimethoprim-Sulfamethoxazole**	**>2/38**	**Resistant**	**<=0.5/9.5**	**Susceptible**	**VME**
APAS_1463	*K. pneumoniae*	Ceftazidime	16	Intermediate	>16	Resistant	mE
		Nitrofurantoin	64	Intermediate	32	Susceptible	mE
APAS_1465	*K. pneumoniae*	Cefazolin	16	Intermediate	8	Susceptible	mE
**APAS_1467**	***E. coli***	**Cefazolin**	**>16**	**Resistant**	**8**	**Susceptible**	**VME**
APAS_1468	*P. mirabilis*	Cefazolin	16	Intermediate	8	Susceptible	mE
APAS_1470	*K. pneumoniae*	Ertapenem	1	Intermediate	<=0.25	Susceptible	mE
**APAS_1473**	***E. coli* (Strain 1)**	**Ampicillin-Sulbactam**	**>16/8**	**Resistant**	**8/4**	**Susceptible**	**VME**
	E. coli (Strain 2)	Ampicillin-Sulbactam	16/8	Intermediate	8/4	Susceptible	mE
APAS_1478	*M. morganii*	Nitrofurantoin	>64	Resistant	64	Intermediate	mE
APAS_1479	*E. coli*	Tobramycin	>8	Resistant	8	Intermediate	mE
APAS_1480	*K. aerogenes*	Nitrofurantoin	64	Intermediate	32	Susceptible	mE
APAS_1488	*P. mirabilis*	Gentamicin	4	Susceptible	8	Intermediate	mE
**APAS_1500**	***E. coli* (Strain 1)**	**Ceftazidime**	**>16**	**Resistant**	**8**	**Susceptible**	**VME**
		**Ertapenem**	**>1**	**Resistant**	**<=0.25**	**Susceptible**	**VME**
		Cefepime	>16	Resistant	8	Intermediate	mE
		**Ampicillin-Sulbactam**	**>16/8**	**Resistant**	**8/4**	**Susceptible**	**VME**
	***E. coli* (Strain 2)**	**Ampicillin-Sulbactam**	**>16/8**	**Resistant**	**4/2**	**Susceptible**	**VME**
APAS_1517	*E. coli*	Tobramycin	4	Susceptible	8	Intermediate	mE
**APAS_1518**	***E. coli***	**Ampicillin**	**>16**	**Resistant**	**<=4**	**Susceptible**	**VME**
		**Cefazolin**	**>16**	**Resistant**	**<=1**	**Susceptible**	**VME**
		**Nitrofurantoin**	**>64**	**Resistant**	**<=16**	**Susceptible**	**VME**
